# A Review of the Quality of Life after Therapeutic Maneuvers in Patients with Benign Paroxysmal Positional Vertigo

**DOI:** 10.22038/IJORL.2021.55574.2912

**Published:** 2021-11

**Authors:** Sertac Yetiser, Ziya Salturk

**Affiliations:** 1 *Department of Otorhinolaryngology Head and Neck surgery , Anadolu Medical Center, Kocaeli 41400, Turkey*

**Keywords:** Benign paroxysmal positional vertigo, Balance, Quality of life, Residual dizziness, Recurrence

## Abstract

**Introduction:**

Benign paroxysmal positional vertigo (BPPV) is a common cause of peripheral vestibular disturbances. Particle repositioning or liberatory maneuvers provide relief of symptoms in the majority of patients. However, studies mainly focus on success. This study aims to review the conditions that may have an impact on residual dizziness or recurrence following therapeutic maneuvers in patients with BPPV.

**Materials and Methods:**

A review of the literature about the analysis of quality of life after therapeutic maneuvers was conducted. Three hundred and seven articles after search in the PubMed database were classified into eight main groups after exclusion of those that are not suitable to predetermined criteria.

**Results:**

Thirty-eight articles for residual dizziness in BPPV, eighty-three articles for the duration of BPPV, forty articles for the type of canal involvement, forty-three articles for the impact of age, one hundred and nine articles for the gender difference, forty-seven articles for co-morbid conditions, one hundred and twenty-four articles for medication and sixty-eight articles for vestibular exercises in BPPV were selected.

**Conclusion:**

VEMP abnormality is a reliable indicator to demonstrate the risk of recurrence. Duration of dizziness has no significant impact on recurrence. But the length of duration is important for residual dizziness. Vestibular rehabilitation or medication alone has no place in treatment but may help to reduce the symptoms in addition to maneuver. Self-perceived evaluation of balance after therapeutic maneuvers is recommended for the selection of those who need rehabilitation or additional medication.

## Introduction

Benign paroxysmal positional vertigo (BPPV) is the most common cause of peripheral vestibular disturbances. Individuals with BPPV have significant functional impairment with a change of head position with respect to gravity (1). BPPV increases the risk of falling in the elder population (2). Predominant etiology is the degeneration of the utricular macula. Particle repositioning or liberatory maneuvers provide relief of symptoms in the majority of patients with BPPV and have a positive impact on the quality of life (QoL) on the physical, functional, and emotional levels. However, some patients may have persistent symptoms or recurrence and can be significantly impaired even after successful repositioning (3,4). Symptoms do not always correlate well with the patients’ restrictions of activities of daily living and limitations of participation. Assessment of perception of disability after maneuvers is as important as the disappearance of positional vertigo. However, studies mainly focus on the success of therapeutic maneuvers. A functional approach that addresses the factors affecting the disability following liberatory or repositioning treatment of BPPV is very few. The underlying causes of residual symptoms affecting the everyday life of those patients need to be explored. This review study aims to provide reliable and valid data about how the management of BPPV with any of the methods used affects the performance of patients on social and work-related issues. This analysis also increases the awareness of the self-perception about the consequences of dizziness in BPPV and allows comparison of different groups of patients with BPPV thus providing sources to improve patient care. 

## Materials and Methods

A review of the literature about the analysis of quality of life (QoL) following therapeutic repositioning or liberatory maneuvers in patients with BPPV was conducted, with data extracted only from articles written in English. The articles were identified through a search in the PubMed database using the keywords *benign paroxysmal positional vertigo*, which yielded 1678 articles. 

Total 347 articles were reviewed for the study after we have excluded those that are technical or case reports, those based on experimental or animal studies, those that focus on the clinic, diagnosis, pathophysiology, etiology, prevalence, incidence, or mechanism other than the outcome and those reports based on surgical treatment. Studies about children or adolescents with paroxysmal positional vertigo and patients with down-beating positional nystagmus or anterior canal BPPV were excluded. Meta-analytic studies on a subject were particularly included provided that the outcome measures were clear. The search only included articles published between 1989 and 2019. If there was more than one article by the same author(s) or institution, only the most recent one matching the criteria and those that were not overlapping were included. The studies were classified into nine main groups: tests or methods used to identify the functional disability, the incidence of residual dizziness, the impact of duration of symptoms, age-related disability, the impact of lateral or posterior canal involvement, gender analysis, investigation of co-morbid conditions, the impact of medication and the impact of physical rehabilitation.

## Results

After searching articles regarding BPPV and nine subjects, the following results were documented, thirty-eight articles were associated with residual dizziness. Eighty-three articles were about the duration of BPPV. Forty articles were about canal involvement of BPPV. Forty-three articles were focused on age in BPPV. One hundred and nine articles were about gender difference. Forty-seven articles investigated co-morbid conditions in BPPV. One hundred and twenty-four articles were about medication (steroids, betahistine, antiemetics, antihistaminics, vestibular suppressants, etc.). Sixty-eight articles were about vestibular exercises in BPPV. The data of the studies analyzing recurrence/residual dizziness associated with comorbid problems in patients with BPPV are summarized in Table I.

## Discussion

**Methods used to identify the disability.** Internationally validated surveys, methods, or instruments to analyze the functional disability in patients with vertigo are “Dizziness Handicap Inventory” (DHI), “Dizzy Factor Inventory” (DFI), “Vestibular Activities and Participation” (VAP) questionnaire, “Vestibular Disorders of Daily Living” scale (VDDL), “Visual Vertigo Analog Scale” (VVAS), “Activities-specific Balance Confidence” (ABC) scale, “Falls Efficacy Scale” (FES), “Vertigo Symptom Scale” (VSS), “Functional Gait Assessment” (FGA), “Dynamic Gait Index” (DGI), “Computerized Dynamic Posturography” (CDP), “Vertigo Handicap Questionnaire” (VHQ), “Subjective Visual vertical” (SVV), “Subjective Visual Horizontal” (SVH), “Vertigo Dizziness Imbalance” questionnaire (VDI) and “European Evaluation of Vertigo” (EEV) (1,5,6,7). 

 The questionnaires are usually administered by post, by e-mail, or are fulfilled by the patients themselves during their control visit. Individual’s perception of their status is important. 

When assessing the patients, it should be kept in mind that people usually report under-scores with remote self-reporting systems compared with face-to-face interviews (5). The proportion of participation of patients invited to remotely participate in the survey is always lower than expected. But it is cost-effective (8).

**Table 1:** The data of the most cited studies analyzing recurrence/residual dizziness associated with comorbid problems in patients with BPPV are presented in the chronological order (PC; posterior canal, LC; lateral canal, SC; superior canal, BPPV; benign paroxysmal positional vertigo, RD; residual dizziness, DHT; Dix-Hallpike test, VAS; visual analog scale, DHI; dizziness handicap inventory, VEMP; vestibular evoked myogenic potentials, CVD; cardio-vascular disease, MD; Meniere disease, DM; Diabetes mellitus, HT; hypertension, HL; hearing loss)

**Article**	**Patients**	**Type of BPPV**	**Methods**	**Control**	**Outcome**	**Insignificant**	**Significant**
Harvey et al, 1994	25	PC	Self-rated evaluation	1 month	Success 68%	Age	Duration
Macias et al, 2000	259	All types	Control DHT and Roll-on tests	6 months	Recurrence 13.5%	Age, gender, trauma	Multiple canals
Nunez et al, 2000	168	PC	Self-rated evaluation	1 week	Recurrence 13.4%	Gender, age, duration, cause	None
Levrat et al 2003	278	PC	DH test	1 week	Recurrence 37.8%	Age, gender,	Duration, traumatic cases
Korres et al 2006	155	All types	DHT, Roll-on test, ENG	2 weeks	Success; 81.2%	Gender, duration	Age, Multiple canals, secondary BPPV, ENG
Seok et al. 2008	49	All types	Interview	3 months	RD 61%	Age, gender, affected side, involved canal	Duration
Choi et al, 2012	120	PC and LC	Control DHT and Roll-on tests	2 weeks	12.5% persistent 10% recurrent	Gender, age,	Recurrent in PC, Persistent in LC
Soto-Varela et al, 2012	412	All types	Control DHT and Roll-on tests	Monthly controls for 6 months	Recurrence at 1^st^ year 14%	Gender, age, duration,	None
Teggi et al 2013	90	All types Idiopathic	VAS and Posturography	From second day	RD 31.1%	Migraine	Duration
Babac et al, 2014	400	All types	Control DHT and Roll-on tests	1 week	Recurrence at one year 15.5%	Duration, HT, DM, migraine, heart disease, CVD, thyroid dysfunction	Age, Head trauma, secondary BPPV, osteoporosis, SC BPPV
Su et al, 2015	243	PC	Control DHT and Roll-on tests	3 months	Recurrence 16.9%	Gender, age, CVD, trauma, Inner ear disease	Sleep disorder
Martellucci et al 2016	97	PC	DHI	At 3 days interval	RD 38.36%	Gender, duration,	Age
Seo et al 2017	44	Idiopathic PC	oVEMP	1 week	RD 60%	Gender, duration, age	VEMP abnormality
Yoon et al, 2018	1426	PC and LC	Control DHT and Roll-on tests	Weekly basis	Recurrence 31.5%	Gender	Age, duration, multiple canal
Luryi et al, 2018	1015	All types	Control DHT and Roll-on tests	A 9-year case review	Recurrence 37%	DM, trauma, MD	Gender (female)
Ding et al, 2019	84	Geotropic type LC	Control DHT, Roll-on tests, DHI and caloric test	1 month for RD, 24 months for recurrence	RD 36%, recurrence 23.8%	Gender, age, duration,	Caloric weakness
Sreenivas et al, 2019	71	PC and LC	Control DHT and Roll-on tests	6 months-1 year follow-up	Recurrence 22.5%	HL, Vit D, Thyroid dysfunction, Cholesterol	Age, DM, hypertension
Zhu et al. 2019	1021	PC and LC	Self-rated evaluation	1 week and 1 year	Recurrence at 1 year 25.2%	Age, duration	Gender, hypertension Migraine, HL, MD, hyperlipidemia

Negative Dix-Hallpike or head-roll maneuvers following treatment do not warrant that BPPV is over since the recurrence is common. Beynon et al have reported that recurrence in 6^th^ months after successful repositioning is around 45% (9). The estimated recurrence rate per year is 15% (10). Surveys are important to understand the patient’s limitations. However, apart from questionnaires in search of disability, evidence-based solid data to exhibit the underlying problem are very few. Studies indicate that vestibular evoked myogenic potentials (VEMP) can be used to demonstrate whether the treatment was successful or not and whether there is a risk of recurrence or not since BPPV in patients with otolithic dysfunction tends to recur (11-13). Persistent abnormality of cVEMP and oVEMP is associated with residual dizziness and poor rehabilitation outcome (14,15). Additionally, VEMP abnormality is more frequent in patients with recurrence than in patients without recurrence (16).

**Incidence of residual dizziness and/or functional disability.**

The success of canalith repositioning maneuvers is high. However, multiple treatment sessions are required in 25-35% of patients. A close correlation between BPPV and functional and emotional impact of the disease has been reported (17).

Questionnaires assessing the functional disability suggest that handicap and distress are quite dense, and perception of handicap is pretty much related to functional performance. BPPV negatively influences everyday tasks and the adverse effect of anxiety on the management of BPPV is correlated with the duration. Anxiety improves 1 week after the treatment at the earliest and recovery continues even at the end of the first month (18). Impairment of postural control is common, but it markedly improves after re-positioning maneuvers (19). On the other hand, around 60% of patients who undergo re-positioning treatment with successful results as demonstrated with normal Dix-Hallpike test after the treatment still experience persistent residual dizziness (4). Residual dizziness may be explained by some theories. First, canalith may incompletely return to the utricle following re-positioning maneuver and a small number of remaining otoconia inside the canal will not provoke nystagmus during control testing but may be enough to cause dizziness. Second, there is probably a persistent utricular degeneration which leads to regular draining of the otoconia to the canals (20). Probably, the re-positioning maneuver is not able to treat what has already been damaged by the otoconia vestibular system, and the cause of dizziness after the repositioning maneuver comes from otolithic dysfunction. 

Another possibility is the co-existence of a hidden vestibular pathology that is difficult to identify from history. Residual dizziness is related to the duration of the disease since longer time may be needed for central adaptation after treatment. Patients with residual dizziness have higher anxiety than patients with no residual dizziness (21). 

**The impact of duration of BPPV.**

Conflicting results have been reported in terms of the impact of the duration of BPPV on recurrence. Some of the clinical studies indicate that the duration of BPPV has no significant impact on recurrence rate (3,10,22,23). 

While some studies indicate its significant impact (4,16,24). However, the length of duration is important for residual dizziness since longer time may be needed for central restoration of postural stability (4,25,26). Patients with a duration of symptoms less than 60 days after the first attack of BPPV have a high dependence on visual input for postural stability. Posturographic analysis indicates that the adaptation mechanisms to restore the sensory conflict between the affected vestibular system and vision take time.

 Therefore, prompt diagnosis and early treatment of BPPV are important. A decrease in disease duration has been reported to be associated with low residual disease and an increase in quality of life (27,28).

**The impact of the canal involvement and the type of BPPV on functional disability.**

 Studies analyzing the impact of canal involvement on the recurrence rate and patient-perceived disability are very few. Martens et al have reported that patients with lateral canal BPPV have increased self-reported disability, lower vitamin D levels, and longer duration of symptoms (29).

Choi et al have found that the recurrence is much common in posterior canal BPPV as compared to lateral canal BPPV (30). However, persistent cases are common in lateral canal BPPV. Studies indicate a significant correlation between the recurrence, functional disability, and simultaneous multiple canal involvement (31,32). 

**Age-related disability.**

BPPV is more common in elder patients. Beyond the acute sense of spinning with rapid head motion it also negatively affects the elder’s gait and balance and increases the risk of falling (2,5). Age is not a risk factor in terms of recurrence (10,22,24,26,30,32). However, aging contributes to delayed recovery of symptoms associated with residual dizziness and postural balance is more affected leading to disability and decreased quality of life (33). Multiple sessions of repositioning may be required. Vestibular suppressants that are commonly prescribed for vestibular problems cause significant side effects for elder patients. Association between BPPV and vitamin D deficiency and osteoporosis indicate that BPPV may share risk factors with other common geriatric conditions (34). Lateral canal cupulolithiasis is more common in elder patients and treatment efficacy is lower as compared to other subtypes of BPPV (35).

**Gender analysis of disability.**

The majority of studies report no significant difference between male and female patients in terms of recurrence (22,24,36,37). Studies indicating gender difference in recurrent rates are few (38). No significant difference between vestibular problem associated anxiety and depression in male and female populations have been reported. However, gender influence in symptom reporting has been observed (39).

**Impact of co-morbidities.**

Recovery following maneuvers in patients with BPPV would be expected to complete in a short term. However, the process of “healing” proceeds under the influence of several factors. Patients with BPPV having comorbid problems, mainly diabetes, hypertension, hyperlipidemia, Meniere’s disease, osteoarthrosis, and osteoporosis have recurrent problems even if they have managed with repositioning maneuvers properly (3,40,44). 

**The impact of medication.**

The efficacy of medication to relieve symptoms and to prevent residual disease following repositioning maneuver is controversial although drugs are proved to improve vestibular microcirculation. Comparative studies are very few. Acar et al have investigated the impact of trimetazidine, betahistine, and gingko biloba in the relief of residual dizziness after successful repositioning maneuvers in patients with BPPV (45). 

There was no statistically significant difference in scores between the patient groups studied with Dizziness Handicap Inventory at first, third and fifth days after repositioning maneuver. However, Guneri and Kustutan found that betahistine therapy in addition to the Epley maneuver had more effective results than the Epley maneuver alone. Vestibular suppressants should be avoided not to reduce the mobility of patients (45). 

**The impact of physical/ vestibular rehabilitation on functional disability.**

Individuals with BPPV tend to restrict head movement and activities to prevent vertigo which imposes physical constraints that lead to functional loss and eventually emotional decline. An exercise-based form of treatment known as “vestibular rehabilitation” or “balance training” is the most effective way of managing vestibular dysfunction by stimulating the vestibular system with the eye, head, and body movements. Initially, every trial will provoke dizziness, but repetition will promote adaptation and compensation process eventually resulting in resolution of symptoms (46).

 A systematic Cochran review indicates that when repositioning maneuvers are combined with vestibular exercises, longer-term functional recovery is more effective than repositioning maneuvers alone (49).

Studies are in favor of the positive contribution of rehabilitation therapy in terms of better QoL scores when compared with medication (48). However, vestibular exercises do not provide an extra benefit in the prevention of recurrence (50).

## Conclusion

VEMP abnormality is a reliable indicator to demonstrate the risk of recurrence. Duration of dizziness has no significant impact on recurrence. But the length of duration is important for residual dizziness since longer time may be needed for central restoration of postural stability. Recurrence is higher in posterior canal BPPV. However, multiple therapeutic sessions are required for lateral canal BPPV. Reports on gender analysis in terms of recurrence indicate no significant difference. Vestibular rehabilitation or medication alone has no place for the treatment of patients with BPPV. However, rehabilitation following repositioning maneuver is important particularly for elder patients for a better quality of life. Changing the sleeping habit and replacement of calcium may reduce the incidence of recurrence. Self-rated scales assessing both vestibular balance and psycho-physiological condition are necessary to evaluate the severity of symptoms. Additional information is provided for the selection of those who may need additional rehabilitation or medication.
